# Synergistic Activity of the HSP90 Inhibitor Ganetespib With Lapatinib Reverses Acquired Lapatinib Resistance in HER2-Positive Breast Cancer Cells

**DOI:** 10.3389/fphar.2021.651516

**Published:** 2021-07-05

**Authors:** Min Ye, Wei Huang, Rui Liu, Yingli Kong, Yang Liu, Xiaole Chen, Jianhua Xu

**Affiliations:** ^1^ School of Pharmacy, Fujian Medical University, Fuzhou, China; ^2^ Fuijan Provincial Key Laboratory of Natural Medicine Pharmacology, Fuzhou, China; ^3^ College of Life Sciences, Fujian Agriculature and Forestry University, Fuzhou, China; ^4^ Fujian Key Laboratory of Drug Target Discovery and Structural and Functional Research, Fuzhou, China

**Keywords:** ganetespib, lapatinib, HER2-Pisitive breast cancer, stat3, antitumor

## Abstract

Lapatinib is an FDA-approved EGFR and HER2 tyrosine kinase inhibitor for the treatment of HER2-positive breast cancer patients. However, its therapeutic efficacy is limited by primary or acquired resistance. In the present study, we established breast cancers cells with acquired lapatinib resistance and investigated the antitumor activity of the second-generation HSP90 inhibitor ganetespib in association with lapatinib in lapatinib-sensitive and -resistant cells. The combination treatment showed synergistic inhibition of HER and the downstream PI3K/Akt and Ras/MEK/ERK pathways, in addition to enhancing induction of early apoptotic cell death and G1 arrest in both parent and lapatinib-resistant cells *in vitro*. The joint administration of ganetespib and lapatinib depleted the aberrant nuclear transcription factor STAT3, a mediator of the cell cycle and apoptosis-related pathways that is probably involved in the lapatinib resistance of HER2-positive breast cancer cells. In conjunctive with the augmented inhibition of tumor growth observed in both SKBR3 and SKBR3-L xenografts compared to monotherapy, our data provide a sound preclinical basis for combination treatment with lapatinib and ganetespib for refractory HER2-positive breast cancer.

## Introduction

Members of the human epidermal growth factor receptor (HER/ERBB) family, including EGFR (HER1), HER2, HER3, and HER4, display tyrosine kinase (TK) activity and play key roles in breast cancer (Citri and Yarden, 2006). Aberrations in HER signaling associated with activation by ligand binding to form homo- or heterodimers Arteaga and Engelman (2014) have been associated with a variety of cellular processes, including survival, proliferation and differentiation in carcinogenesis (Zaczek et al., 2005; Linggi and Carpenter, 2006). Both EGFR and HER2 have been unanimously recognized as prognostic biomarkers in clinical therapies, and HER2 amplification accounts for approximately 25% of breast cancers associated with aggressive disease and poorer survival (Tebbutt et al., 2013; Figueroa-Magalhães et al., 2014). Although HER2 has no known ligand, it prefers to homodimerize or heterodimerize with other HER family receptors (Canonici et al., 2013); in particular, the HER2/HER3 dimer is considered to be the most potent in terms of tyrosine phosphorylation and activation of downstream PI3K/Akt and Raf/MEK/ERK signaling (Lee-Hoeflich et al., 2008; Huang et al., 2015). HER4 is not frequently overexpressed in breast cancer, and the oncogenic and tumor-suppressive functions of HER4 remains controversial (Gullick, 2003; Qiu et al., 2008).

Multiple agents have been developed for the treatment of HER2-positive (HER2 + ) breast cancer. Approved HER2-targeted therapies include monoclonal antibodies, such as trastuzumab, pertuzumab, and trastuzumab-DM1 (an antibody drug conjugate), and small molecule tyrosine kinase inhibitors (TKIs), such as lapatinib (Tykerb/GW572016). Lapatinib is an orally available small molecule which blocks both EGFR and HER2 receptors by interacting with the receptor ATP binding site (Xia et al., 2002). It has shown dramatic therapeutic effects, both alone and in combination, for the treatment of breast cancer, including reduction of trastuzumab-refractory risk and use in combination with capecitabine for the treatment of advanced HER2 + breast cancer (Geyer et al., 2006; Nahta et al., 2007).

Over the past decades, HER2-target drugs have shown encouraging outcomes for patients with HER2 + breast cancer. However, resistance to these agents has posed a great challenge to their therapeutic efficacy. In the case of lapatinib, despite advances in our understanding of aggressive HER2 + breast cancer, the inevitable development of primary and acquired resistance dramatically limits its clinical efficiency (Campone et al., 2011). In fact, the medium duration of lapatinib treatment is less than 1 year, and most patients eventually lose responsiveness to lapatinib under HER2-targeted treatment (Blackwell et al., 2009; Blackwell et al., 2010). Therefore, resistance in HER2 + breast cancer has attracted worldwide attention in recent years. Although the precise mechanisms of resistance remain to be clarified, multiple pathways have been reported to be related to resistance, including crosstalk between HER signaling and the persistent activation of PI3K/Akt and Raf/MEK/ERK pathways (Jegg et al., 2012; Shi et al., 2016).

HSP90 is the most abundant molecular chaperone in mammals, and it is expressed at higher levels in malignant cells than in normal cells (Banerji, 2009). It is essential for correct folding, activation and stabilization of more than 200 client proteins, including kinases, hormone receptors and transcription factors (Trepel et al., 2010). Most HSP90 client proteins, such as HER family members, Akt, STAT3, Cyclins, and Cyclin-dependent kinases (CDKs), are involved in critical signaling pathways necessary for cell survival and proliferation (Solit and Chiosis, 2008). HER2 is regarded as one of the client proteins most sensitive to HSP90 inhibition (Citri et al., 2004). For example, the first generation HSP90 inhibitor Geldanamycin and its derivative 17-AAG target HSP90 by binding to the N-terminal ATP pocket (De Mattos-Arruda and Cortes, 2012). Other non-geldanamycin HSP90 inhibitors, such as NVP-AUY922 Jensen et al. (2008) and FW-04-806 (Huang et al., 2014), dissociate HSP90 from its co-chaperones p23 and CDC37, respectively, and result in HER2 ubiquitination and proteasomal degradation through HSP90 inhibition. Ganetespib (STA-9090) is a resorcinolic triazolone small molecule HSP90 inhibitor and has shown significant inhibition in a xenograft model of HER2 + breast cancer with a favorable safety profile (Friedland et al., 2014). Although the objective response rate (ORR) of ganetespib has not yet met the prespecified criteria of the phase Ⅱ open-label study for metastatic breast cancer (MBC), it showed positive clinical activity for trastuzumab-refractory HER2-positive and triple-negative breast cancers (Jhaveri et al., 2014). In addition, results from a phase I trial of ganetespib in combination with paclitaxel and trastuzumab for HER2 + MBC showed promising results, and a phase Ⅱ trial studying trastuzumab-refractory HER2 + MBC is currently in the planning stages (Jhaveri et al., 2016).

Combined treatment with different HER2-targeted agents with complementary mechanisms has been widely adopted in the treatment of breast cancer and has proven to be a robust approach to prevent or delay resistance (Baselga et al., 2012; Oude Munnink et al., 2012). The aim of the present study is to investigate the antitumor activity of ganetespib in combination with lapatinib on HER2 + breast cancer cells, as well as the anti-resistance activity of ganetespib alone and in combination with lapatinib in lapatinib-resistant cell lines. Herein, we show that a novel combination of HER2-targeted therapies, ganetespib and lapatinib, exhibits synergistic inhibition of HER signaling and the downstream PI3K/Akt and Ras/MEK/ERK pathways in both lapatinib-sensitive and resistant HER2 + breast cancer cells *in vitro*. Moreover, joint administration of ganetespib and lapatinib not only induced enhanced early apoptosis and G0/G1 arrest, but also depleted the aberrant nuclear transcription factors STAT3 and its downstream signaling partners, thought to be associated with the mechanism of lapatinib resistance in HER2-positive breast cancer cells. Furthermore, the combination of ganetespib and lapatinib treatments enhanced inhibition of tumor growth in both SKBR3 and SKBR3-L xenografts compared to monotherapy, suggesting a promising therapeutic strategy for HER2 + breast cancer.

## Materials and Methods

### Cell lines, plasmid and Reagents

SKBR3 and BT474 cell lines were obtained from the American Type Culture Collection (ATCC). SKBR3 and BT474 cells were cultured in RPMI-1640 medium. Lapatinib-resistant cell lines (SKBR3-L and BT474-L) were established by treating cells with increasing concentrations of lapatinib (0.25–5 µM) for a period of 6 months. Single-cell clonal populations were obtained from a pool of resistant cells, and cells were expanded in RPMI-1640 medium in the presence of 2 µM lapatinib. All cells were maintained under standard cell culture conditions at 37°C and 5% CO_2_ in a humid environment.

Lapatinib was obtained from LC Laboratories (Woburn, MA, United States). Ganetespib was purchased from MedChem Express (NJ, United States). MTS was purchased from Promega. Primary antibodies against Akt and β-actin were obtained from Santa Cruz Biotechnology. Other primary antibodies against p44/42 MAPK (ERK1/2), Phospho-p44/42 MAPK (T202/Y204) (p-ERK), Phospho-Akt (Thr308), STAT3, Bcl-2, Bcl-xl, and c-Myc were obtained from Cell Signaling Technology. The HER/ErbB family Antibody Sample Kit from Cell Signaling Technology included antibodies against EGFR (D38B1), HER2/ErbB2 (D8F12), HER3/ErbB3 (D22C5), HER4/ErbB4 (111B2), Phospho-EGFR (Tyr1068), Phospho-HER2/ErB2 (Tyr1221/1222), Phospho-HER3/ErB3 (Tyr1289), and Phosphor-HER4/ErbB4 (Tyr1284). Fluorescein isothiocyanate (FITC) Annexin-V Apoptosis Detection Kit was purchased from BD Biosciences. Other chemical reagents were obtained from Sigma Aldrich.

### Cell Viability Assays

Cells (5 × 10^3^/well) were seeded in 96-well plates and treated with escalating doses of lapatinib (ranging from 0.05 to 80 µM) and ganetespib (ranging from 0.01 to 0.16 µM) as single agents or in combination. After treatment for 48 h, cell viability was assessed by MTS assay (Promega) according to the manufacturer’s instruction. Results were calculated based on the assumption that the number of living cells was proportional to the absorbance at 490 nm, and results are presented as means ± SD deviation from three independent experiments. Inhibition graphs are based on mean values obtained from each concentration relative to control values, and half-maximal inhibitory concentrations (IC_50_) were calculated using PASWstatistics 18 (SPSS, Inc).

### Luciferase Assay

Plasmid STAT3-TAL-Luc (Addgene plasmid # 46933), a gift from Afshin Dowlati described in (Dabir et al., 2009), was transfected into SKBR3, SKBR3-L, BT474, and BT474-L cell lines using Lipofectamine 2000 reagent (Invitrogen) according to the manufacturer’s instruction. Stable clones that showed high luciferase activity were selected for the luciferase assay and measured using the Promega luciferase kit.

### siRNA Study

SKBR3-L and BT474-L cells were transfected with STAT3 siRNA (Santa Cruz-sc-29493) or control siRNA (sc-37007) using Lipofectamine 2000 (Invitrogen) according to the manufacturer’s instructions. Cell lysates were collected for western blot after 24 h of treatment.

### Preparation of Cell Lysates and Cell Fractions

For whole cell lysates, 1 × 10^7^ cultured cells were harvested and washed twice with ice-cold PBS, then lyzed for 15 min at 4°C with 500 µL lysis buffer (10 mM Tris-HCl pH 8.0, 1 mM EDTA, 2% sodium dodecyl sulfate (SDS); 5 mM dithiothreitol (DTT); 10 mM phenylmethanesulfonyl fluoride (PMSF, Sigma Aldrich), a cocktail of protease and phosphatase inhibitors (Roche, Indianapolis, IN), and PhosSTOP (Roche Diagnostics). Samples were then centrifuged at 12,000 rpm for 10 min, after which the supernatant was collected and stored at -70°C until later use.

For the preparation of cytoplasmic and nuclear factions, 1×10^7^ cultured cells were washed with PBS and suspended in 200 µL of lysis buffer (10 mM HEPES, pH 7.9; 10 mM KCl; 0.1 mM EDTA; 0.1 mM EGTA; 1 mM DTT; 0.5 mM PMSF and protease inhibitor cocktail). Cells were incubated on ice for 15 min, after which 6.5 µL of 12.5% NP-40 were added; the contents were mixed, then centrifuged for 1 min at 12,000 rpm. The supernatant was saved as the cytoplasmic fraction. The pellet was resuspended in 12.5 µL of ice-cold nuclear extraction buffer (20 mM HEPES, pH 7.9; 0.4 M NaCl; 1 mM EDTA; 1 mM EGTA; 1 mM DTT; 1 mM PMSF and protease inhibitor cocktail) and incubated on ice for 40 min, with mixing every 10 min. The solution was then centrifuged at 12,000 rpm for 5 min at 4°C. The resulting supernatant was saved as the nuclear fraction.

### Western Blot Analysis

Protein concentration was determined using the BCA Protein Assay Kit (Thermo Scientific) according to the manufacturer’s instructions. Equal amounts of protein were separated using SDS-PAGE, transferred to PVDF membranes and blotted with specific primary antibodies. Proteins were detected *via* incubation with horseradish peroxidase-conjugated secondary antibodies and visualized with SuperSignal WestPico (Thermo Scientific). All western blots were performed at least three times to ensure replicability.

### Apoptosis Assay

Apoptosis was assayed using the FITC Annexin-V Apoptosis Detection Kit I (BD Biosciences) according to the manufacturer’s instructions. Briefly, HER2 + breast cancer cells were treated with a vehicle control (DMSO), lapatinib, ganetespib or lapatinib plus ganetespib for 24 h. Cells were subsequently stained with FITC and PI for 15 min at room temperature and analyzed by flow cytometry.

### Tumor Xenograft

BALB/c (nu/nu) athymic mice were purchased from Shanghai Institutes for Biological Sciences, Chinese Academy of Sciences. For xenografts, 6 mm^3^ tumor fragments were implanted into the subcutaneous tissue of the axillary region using a trocar needle. Mice were randomly assigned to groups (n = 6) when their tumor burdens reached approximately 50 mm^3^. Animals were treated with ganetespib for 2 days/wk (in a vehicle of 1% DMSO, 30% polyethylene glycol and 1% Tween 80, intraperitoneal injection) and lapatinib for 7 days/wk (in a vehicle of 1% DMSO, 30% polyethylene glycol and 1% Tween 80, oral gavage). Tumor volumes were calculated using the following ellipsoid formula [D × (d2)]/2, in which D is the large diameter of the tumor, and d is the small diameter. Tumor volumes are plotted as means ± SD. All animal experiments were approved by the Animal Care and Use Committee, Fujian Medical University, China.

### Immunohistochemistry

Tumor sections were fixed in 4% paraformaldehyde for 24 h at room temperature before dehydration and paraffin-embedding. After antigen retrieval and incubation with hydrogen peroxide, tumor sections were incubated with primary antibodies against HER2. Sections were sequentially incubated with secondary antibody and horseradish peroxidase conjugated with polymer for 30 min. Contrast was applied with hematoxylin, and sections were mounted in Canadian balsam and scanned by Olympus 1X73.

### Statistical Analysis

ANOVA was employed for comparisons across multiple groups. The data are reported as mean ± SD (n = 3 per group). Statistical analysis was performed using PASWstatistics 18 (SPSS, Inc); *p*＜0.05 was considered as the cutoff for statistical significance.

## Results

### Potency of Ganetespib in Lapatinib-Sensitive and Resistant HER2+ Breast Cancer Cells

**FIGURE 1:**
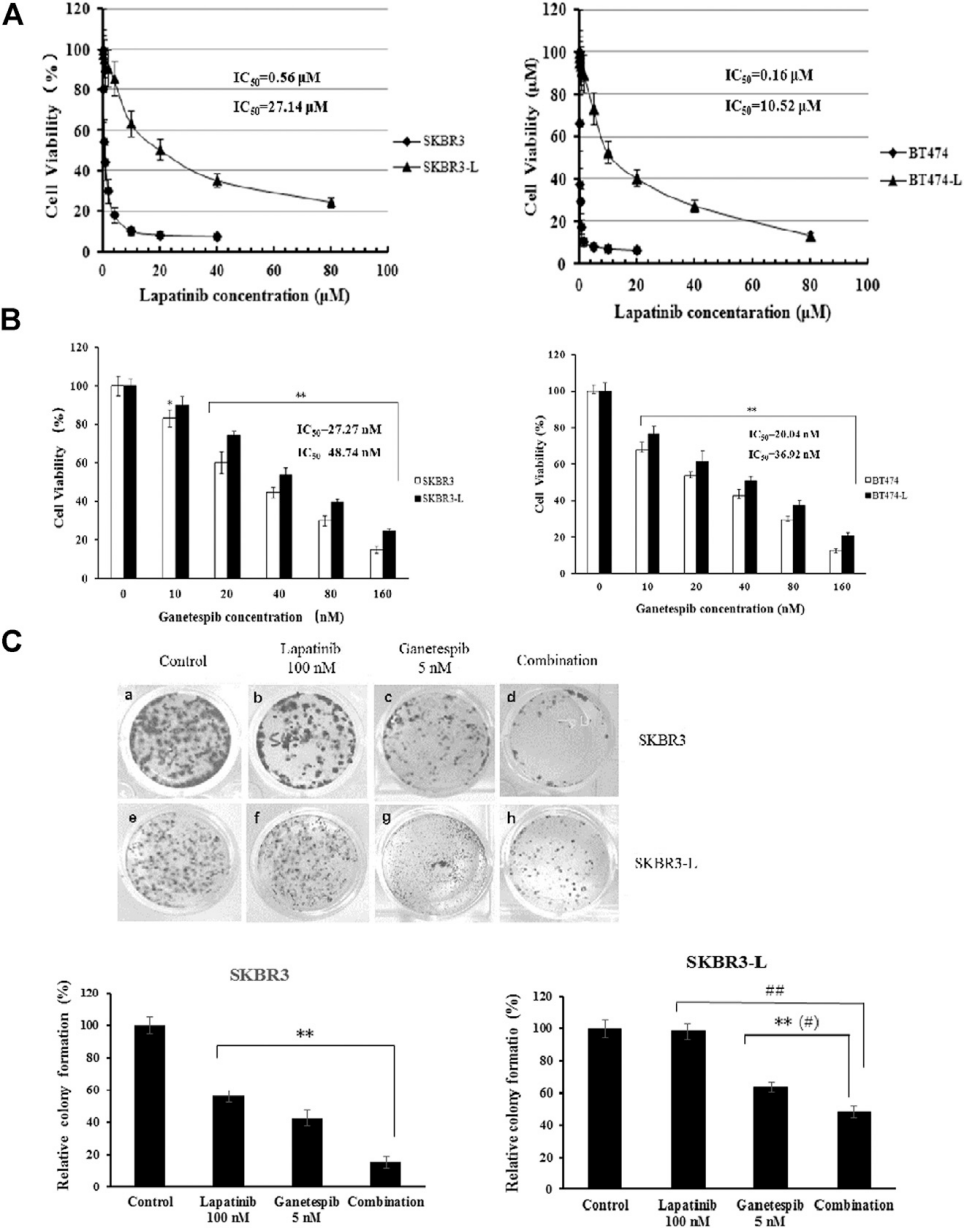
Antiproliferative activity of ganetespib in lapatinib-sensitive and -resistant HER2 + breast cancer. **(A)** SKBR3, SKBR3-L, BT474, and BT474-L were treated for 48 h in the presence of lapatinib or DMSO vehicle to confirm the lapatinib-resistance of SKBR3-L and BT474-L breast cancer cell lines. **(B)** SKBR3, SKBR3-L, BT474, and BT474-L were treated for 48 h with ganetespib or DMSO vehicle. Cell viability was measured with MTS and is expressed as a percentage of vehicle-treated control. Results are presented as means ± SD of three independent experiments. *p < 0.05: significant difference from control by ANOVA; **p < 0.01: very significant difference from control by ANOVA. **(C)** Effect of ganetespib and lapatinib on colony formation of SKBR3 and SKBR3-L cells. **p < 0.01 *vs.* control, # *p* < 0.05 and ## *p* < 0.01 *vs*. combination administration by ANOVA.

The isolation and characterization of lapatinib-conditioned HER2-overexpressing breast cancer cell lines SKBR3 and BT474 is described in Materials and Methods. Lapatinib-resistant cells SKBR3-L and BT474-L cells were obtained according to cell proliferation assay results. The data confirmed that SKBR3-L and BT474-L were refractory to lapatinib *in vitro*, with IC_50_ values increased approximately 48 and 66 -fold in the SKBR3-L and BT474-L lines, respectively, compared to their parent cells (Figure 1A). To confirm our results, a colony formation assay was performed using SKBR3 and SKBR3-L cells treated with lapatinib for 14 days. The results showed that lapatinib-resistant cells showed stronger drug resistance to lapatinib than the parent cells (Figure 1C).

The proliferation-inhibitive effect of ganetespib on both lapatinib-sensitive and -resistant cells was assessed *via* cell viability and colony formation assays. Results showed that SKBR3-L and BT474-L cells were slightly less sensitive than the parent cells to treatment with ganetespib (Figure 1B). Consistent with the results of the cell viability assay, more colony formation was observed for the SKBR3-L cells after ganetespib treatment compared with to the SKBR3 cells (Figure 1C). A similar result was observed for BT474 and BT474-L cells (data not shown). Collectively, ganetespib displayed a robust antiproliferative effect on both lapatinib-sensitive and -resistant HER2 + breast cancer cells.

### Synergistic Effect of Ganetespib and Lapatinib in Lapatinib-Sensitive and Resistant HER2 + Breast Cancer Cells and HER Signaling

**FIGURE 2:**
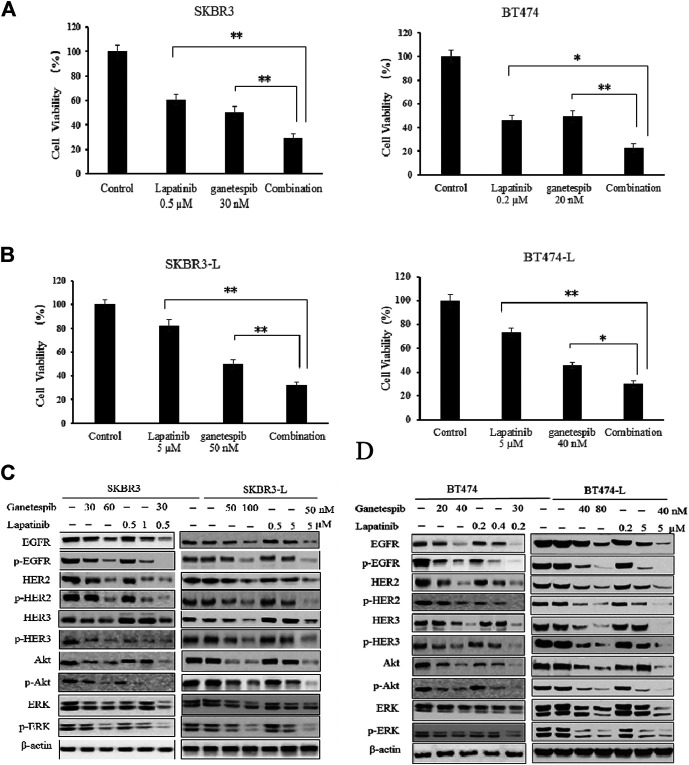
Synergistic effect of ganetespib and lapatinib in lapatinib-sensitive and -resistant breast cancer cell lines. **(A)** SKBR3, BT474, **(B)** SKBR3-L, and BT474-L cells were treated with ganetespib, lapatinib or ganetespib plus lapatinib at the indicated optimal combinative concentrations obtained from the Chou-Talalay method (CompuSyn software). Data are presented as means ± SD of three independent experiments, *p < 0.05, **p < 0.01 *vs*. combination administration for each cell line by ANOVA. **(C)** SKBR3, SKBR3-L, **(D)** BT474, and BT474-L cells were incubated with ganetespib, lapatinib or combination for 24 h. Protein levels were analyzed by western blot for EGFR, HER2, HER3, Akt, ERK and their phosphorylated forms.

The combination of different HER2-targeted agents with complementary mechanisms has proven to be a robust approach to enhance the efficacy of therapies for HER2 + breast cancer (Figueroa-Magalhães et al., 2014; Elster et al., 2015). The feasibility of combining ganetespib with lapatinib was assessed through a series of proliferation assays. First, we examined the single and additive effects of the compounds on proliferation of SKBR3 and BT-474 cell lines treated with ganetespib (ranging from 0.01 to 0.16 μM) and/or lapatinib (ranging from 0.05 to 10 μM). Potential synergy between ganetespib and lapatinib was evaluated by the Chou–Talalay method, which is widely employed in drug combination and synergy quantification (Chou, 2010), The resulting combination index (CI) theorem offers quantitative definitions for additive effect (CI = 1), synergy (CI < 1), and antagonism (CI > 1) of drug combinations. The effects of the optimal combinatorial concentrations obtained from the Chou–Talalay method on cell viability was tested *via* MTS assay. Results from Figure 2A show significantly synergistic inhibition of proliferation by the drug combinations in both SKBR3 (CI = 0.59) and BT474 (CI = 0.53) cell lines, compared with single drug treatments (*p* < 0.05, Figure 2A). The synergistic inhibition of lapatinib plus ganetespib was also affirmed by colony formation assay (Figure 1C). Notably, a greater inhibitory effect on colony formation had also been observed in the SKBR3-L cells following combination treatment, compared to treatment with a single agent (*p* < 0.05), indicating the superior ability of combination treatment to overcome acquired lapatinib resistance. To better understand the action of lapatinib in lapatinib-resistant cells, we selected the concentration of lapatinib to be 5 µM for the cell proliferation assay, approximately 10–25 fold higher than the dose received by their parental cells. Surprisingly, the high-dose lapatinib treatment only slightly suppressed cell proliferation in the SKBR3-L and BT474-L cells (*p* < 0.05). However, treatment in combination with ganetespib had a synergistic effect on both resistant cell lines (Figure 2B).

Continued activation of HER-mediated signaling pathways has been reported as a common feature of lapatinib resistance (Shi et al., 2016); therefore, we assessed the effects of ganetespib and lapatinib, alone or in combination, on critical elements of these pathways. In both SKBR3 and BT474 cells, dose-dependent dephosphorylation of EGFR, HER2, HER3, Akt, and ERK has been observed following 24 h of treatment with lapatinib and ganetespib. Although total EGFR and HER2 were reduced, the persistent activation of Akt and ERK correlated with the upregulation of upstream HER3 induced by lapatinib, consistent with other reports (Xia et al., 2013; Leung et al., 2015). No changes to HER4 and phospho-HER4 (p-HER4) were detected due to its low expression (data not shown). Ganetespib therapy resulted in decreased expression of both total and phosphorylated forms of HER family proteins, as well as downstream oncoproteins Akt and ERK. The combination of ganetespib and lapatinib enhanced the reduction of EGFR and HER2, while the induction of HER3 and downstream Akt and ERK was abrogated by the additive effect (Figure 2C).

Drug treatments alone and in combination were also tested on the lapatinib-resistant cell lines SKBR3-L and BT474-L to test the effects of ganetespib and lapatinib, alone and in combination, on acquired lapatinib resistance (Figures 2C,D). The results showed that lapatinib treatment could still reactivate expression of HER3 and downstream Akt and ERK in lapatinib-resistant cells, while single-agent treatment with lapatinib or ganetespib at higher concentrations could also downregulate the phosphorylated forms of HER receptors and downstream Akt and ERK expression. Combination treatment could also trigger synergistic inhibition in both SKBR-L and BT474-L cell lines.

### Effect of Ganetespib and Lapatinib Alone or Combination on Cell Cycle and Apoptosis

Since the major difference between the lapatinib-sensitive and -resistant cells is cell death caused by lapatinib treatment, we used flow cytometry analysis to assess the effects of lapatinib and ganetespib, alone or combined, on cell apoptosis and cell cycle distribution.

**FIGURE 3:**
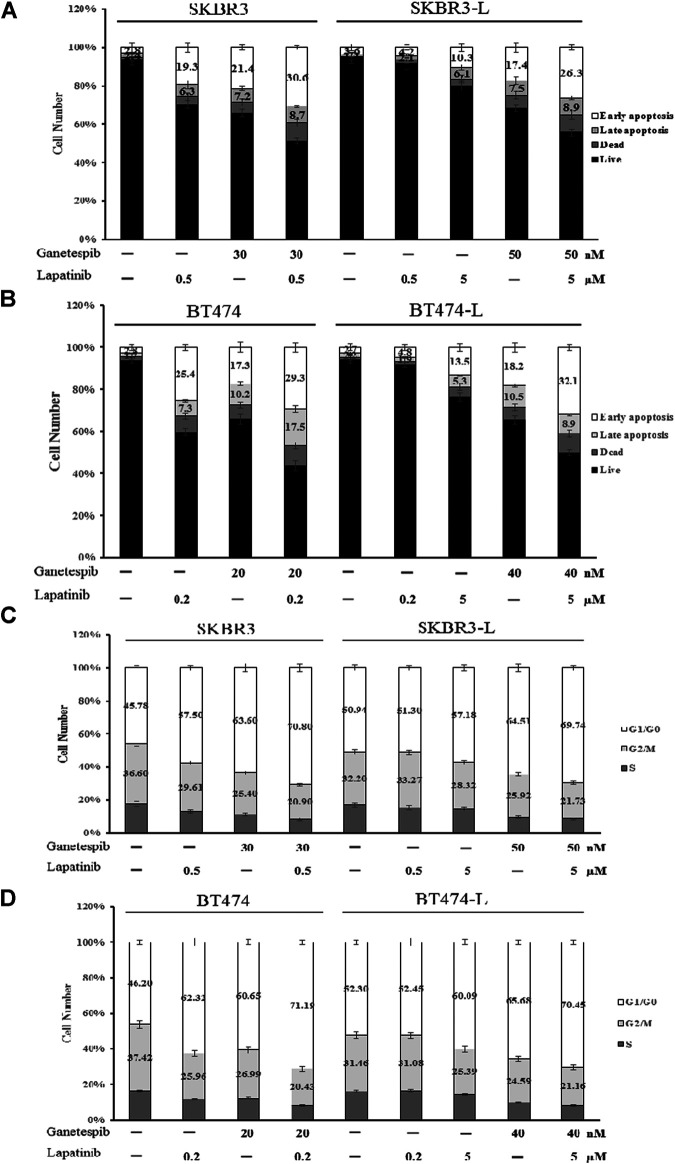
Effect of ganetespib and lapatinib, alone or in combination, on cell cycle and apoptosis. **(A)** SKBR3, SKBR3-L, **(B)** BT474, and BT474-L cells were treated with ganetespib, lapatinib or combination for 24 h. Apoptotic cell death was detected by staining cells with Annexin-V: FITC Apoptosis Detection Kit before flow cytometry analysis. **(C)** SKBR3, SKBR3-L, **(D)** BT474, and BT474-L cells were treated with ganetespib and lapatinib, alone or in combination, for 24 h. The cells were then fixed with 70% ethanol at –20°C overnight, incubated with RNase A at 37°C for 30 min, stained with propidium iodide for 10 min, and analyzed *via* flow cytometry.

Apoptotic cell death analysis was performed using the FITC: Annexin-V Apoptosis Detection Kit I. Both lapatinib and ganetespib treatment resulted in a notable early apoptotic cell population compared to untreated control cells, and combination treatment caused an even more significant increase in early apoptosis, demonstrating that joint administration can induce cell death *via* apoptosis in both lapatinib-sensitive and -resistant breast cancer cell lines (Figures 3A,B).

The effects of lapatinib, ganetespib and the combination on cell cycle progression in SKBR3, BT474 and lapatinib-resistant cell lines were also determined. Both lapatinib and ganetespib treatments resulted in a significant increase of the proportion of cells in the G0/G1 phase, as well as a corresponding decrease in S and G2/M phases. The combination of lapatinib and ganetespib generated a G0/G1 increase and S-phase reduction compared with control cells in both parental and lapatinib-resistant SKBR3 and BT474 cells (Figures 3C,D).

Notably, the cell population distributions were similar in untreated parental and resistant cells, and higher concentrations of lapatinib and ganetespib were required for apoptosis induction and cell cycle arrest in SKBR3-L and BT474-L cells, demonstrating that escape from early apoptosis and G0/G1 arrest probably represents a major means of lapatinib resistance in breast cancer. Therefore, the principle of anti-resistance by ganetespib and combination treatment may lie in enhanced apoptosis induction and cell cycle arrest.

### Suppression of STAT3 Mediated Pathway and Reversion of Lapatinib Resistance by Ganetespib and Lapatinib Combination

To ensure that genomic content is correctly transmitted to the next generation, a series of surveillance pathways are activated to control cell-cycle transitions, DNA replication, DNA repair and apoptosis (Abraham, 2001). One major pathway known to couple the DNA damage cell cycle check point and the apoptosis pathway is the signal transducer and activators of transcription (STAT) pathway, which includes STAT3 (Stephanou and Latchman, 2005). The STAT3 transcription factor is well known to function as an anti-apoptotic factor. It can be activated by tyrosine kinase signaling, such as from the HER pathways Vigneron et al. (2008), and is accompanied by increased expression of important downstream cell cycle and survival regulators, including cyclin D1, Bcl-xl, CDK4, and c-Myc (Inghirami et al., 2005). Moreover, aberrant signaling by STAT3 proteins has been demonstrated to play important roles in the establishment of resistance to tyrosine kinases inhibitors (Sen et al., 2012). Therefore, we investigated STAT3-related signaling to determine its involvement in the mechanism of lapatinib resistance.

**FIGURE 4:**
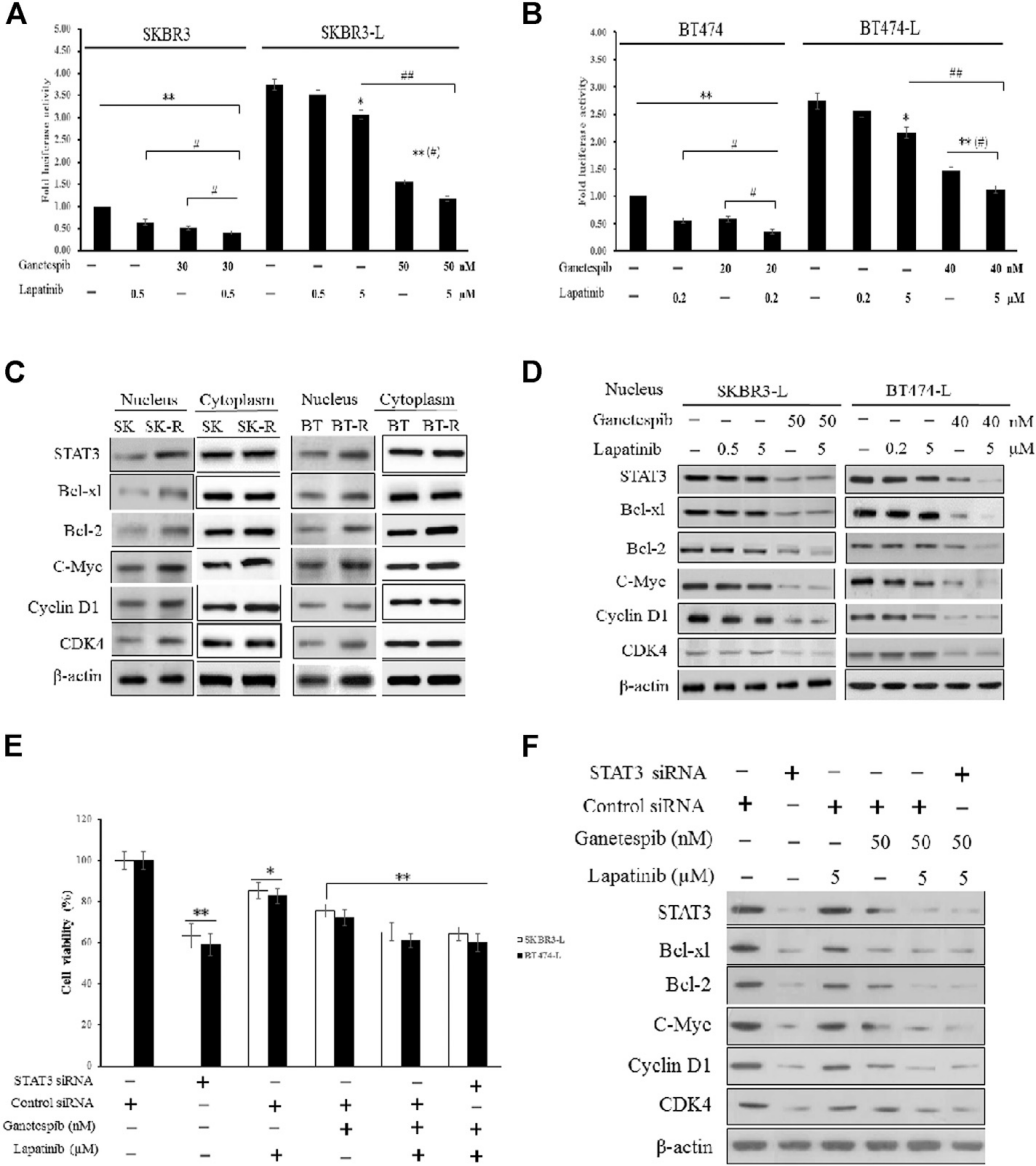
Suppression of STAT3-mediated pathways and reversion of lapatinib resistance by combination treatment with ganetespib and lapatinib. **(A)** SKBR3, SKBR3-L, **(B)** BT474, and BT474-L were transfected using the chromosomally integrated luciferase reporter plasmid STAT3-TAL-Luc. Cells were treated with ganetespib, lapatinib or both for 24 h. Firefly luciferase activity was measured by Varioskan Flash (Thermo Scientific). Results are shown as the mean fold induction over the activity in untreated cells. Data shown are representative of mean ± SD of three independent experiments. *p < 0.05, **p < 0.01 *vs*. control by ANOVA. **(C)** Cytoplasmic and nuclear expression of STAT3, Bcl-xl, Bcl-2, C-Myc, Cyclin D1, CDK4 by western blot, using β-actin as loading control. **(D)** SKBR3-L and BT474-L cells were treated with ganetespib, lapatinib or combination for 24 h, and STAT3-mediated nuclear signaling proteins were detected *via* western blot. **(E)** SKBR3-L and BT474-L cells were treated with STAT3 siRNA or control siRNA, then incubated with ganetespib, lapatinib or both for 48 h to test cell viability. *p < 0.05, **p < 0.01 *vs*. control siRNA transfected cells by ANOVA. **(F)** SKBR3-L cells transfected with STAT3 siRNA or control siRNA and treated with ganetespib, lapatinib or combination for 24 h. Nuclear fractions were analyzed *via* western blot using antibodies against STAT3, Bcl-xl, Bcl-2, C-Myc, Cyclin D1, and CDK4.

First, we constructed SKBR3, SKBR3-L, BT474, and BT474-L cell lines harboring a chromosomally integrated luciferase reporter plasmid driven by STAT3 response elements from the TRAIL promotor (Dabir et al., 2009), with the goal of gaining insight into the effects of lapatinib and ganetespib, alone or in combination, on transcriptional activity. Results showed that STAT3 transcriptional activity was significantly higher in lapatinib-resistant cells, with luciferase activity increased more than 2-fold in SKBR3-L and BT474-L cells compared to their parent cells (Figures 4A,B). STAT3 transcriptional activity could be suppressed in the resistant cells by high-dose lapatinib, ganetespib or combination treatment, and the combination led to greater inhibition of the transfected STAT3 promoter-reporter compared to the single drug treatments (*p* < 0.05).

Since STAT3 is an essential transcription factor involved in nuclear transportation of cell cycle and survival regulators, we also performed western blots to assess cytoplasmic and nuclear protein levels of both lapatinib-sensitive and -resistant cells. Although there were no significant differences in cytoplasmic protein levels, higher nuclear expression of STAT3, as well as associated factors involved in Bcl-2, Bcl-xl, C-Myc, CDK4, and Cyclin D signaling, was observed in lapatinib-resistant cells compared to sensitive cells (Figure 4C). Treatment of lapatinib-resistant SKBR3-L and BT474-L cells with ganetespib and lapatinib, alone or in combination, reduced nuclear STAT3-mediated signaling (Figure 4D). To further clarify the effects of single agent treatments on the STAT3 survival pathway, we knocked down STAT3 in SKBR3-L and BT474-L cells using siRNA. Results showed that the cell death proportion observed in combination-treated lapatinib-resistant cells was approximately equal to that observed in STAT3-depleted cells (*p* > 0.05), but less than that induced by treatment with ganetespib or lapatinib alone (Figure 4E). Similar results were observed for BT474-L cells (data not shown). Importantly, combined administration could not induce further cell death after STAT3 knockdown (*p* > 0.05), suggesting that the STAT3-mediated survival pathway may be involved in lapatinib resistance and that the reversal of resistance by combined treatment likely occurs through STAT3 pathway inhibition.

### Antitumor Activity of Ganetespib and Lapatinib Alone or Combination in Xenograft Models *in vivo*

**FIGURE 5:**
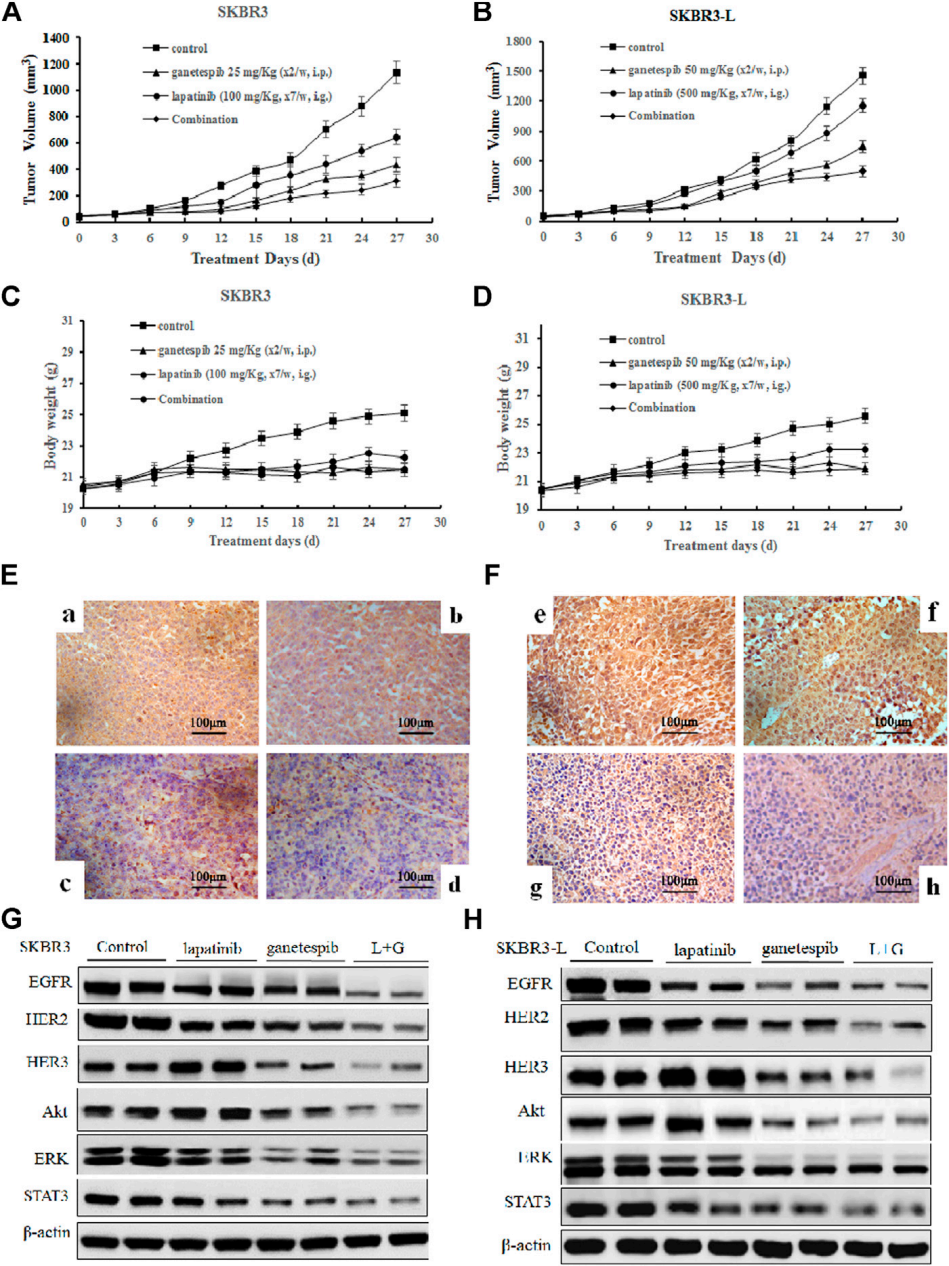
Antitumor activity of ganetespib and lapatinib, alone or in combination, in xenograft models *in vivo*. **(A)** and **(B)** Bearing tumors with volumes of approximately 50 mm3, SKBR3 and SKBR3-L tumor xenograft nude mice were randomized into treatment groups (n = 6/group) and received vehicle, lapatinib, ganetespib or combination (in a vehicle of 1% DMSO, 30% polyethylene glycol and 1% Tween 80) for 4 weeks. Data are presented as means ± SD (n = 6, *p* < 0.05). **(C)** and **(D)** Mouse body weight was measured every three days. HER2 expression in **(E)** SKBR3 and **(F)** SKBR3-L tumors was determined by immunohistochemistry. Pictures a and e show vehicle controls; pictures b and f, c and g, d and h show lapatinib-, ganetespib-, and combination-treated groups for the SKBR3 and SKBR3-L xenografts, respectively. Tumor tissues excised from **(G)** SKBR3 and **(H)** SKBR3-L xenografts were lyzed, and changes in the levels of EGFR, HER2, HER3, Akt, ERK, and STAT3 protein were assessed by western blot.

In order to assess the potential therapeutic benefit of combining ganetespib with lapatinib *in vivo*, we employed SKBR3 and SKBR3-L xenograft models and observed the antitumor effects of ganetespib, lapatinib and their combination. SKBR3 xenografts were more sensitive to lapatinib than SKBR3-L xenografts. Lapatinib, ganetespib and their combination decreased tumor growth at respective rates of 43.4% (*p* = 0.024), 61.8% (*p* = 0.018) and 72.6% (*p* = 0.007) for SKBR3 xenografts, compared to 17.6% (*p* = 0.035), 55.8% (*p* = 0.017) and 65.9% (*p* = 0.009) for SKBR3-L xenografts, suggesting that combined treatment with lapatinib and ganetespib enhanced inhibition of tumor growth compared to monotherapy (Figures 5A,B). All treatment groups survived without appreciable adverse effects on body weight loss or other toxic signs (Figures 5C,D). Immunohistochemical analysis of ER2 oncoproteins from tumor tissues showed a more significant decrease following treatment with lapatinib coupled with ganetespib than following single agent treatment for both SKBR3 and SKBR3-L tumor tissues (Figures 5E,F). Protein levels of HER signaling pathway members and STAT3 in tumor tissues showed a synergistic decrease in EGFR, HER2 and STAT3, and the activation of HER3 and downstream signaling effectors Akt and ERK was abrogated by joint treatment in both SKBR3 and SKBR3-L xenografts. These data are consistent with the results obtained in cellular studies, corroborating the synergistic benefit of lapatinib and ganetespib in treatment of lapatinib-refractory HER2 + breast cancer.

## Discussion

In the previous decade, treatment of HER2 + breast cancer has been significantly improved by the employment of HER2-targeted monoclonal antibodies and TKIs. However, resistance to these agents has greatly limited their therapeutic efficacy (Nahta et al., 2006; Arteaga et al., 2011). However, the introduction of HSP90 inhibitors might renew hope for refractory HER2+ breast cancer patients. HSP90 plays a key role in regulating the stability, maturation, and activation of a wide range of client substrates, including HER family members, STAT family members, Akt, cyclins and CDKs, that are essential for cancer cell survival and proliferation (Kamal et al., 2003; Kamal et al., 2004; Falsone et al., 2005; Zhao et al., 2005). Among these factors, HER2 has been identified as one of the oncogenes most sensitive to HSP90 inhibition. Several HSP90 inhibitors have been reported as effective against refractory HER2 + breast cancer. For example, a phase I trial of the first-generation HSP90 inhibitor 17-AAG in combination with trastuzumab has shown a positive profile in trastuzumab-refractory HER2-overexpressing breast cancer (Modi et al., 2007; Raja et al., 2008). The HSP90 inhibitor IPI-504 shows antitumor activity alone and when combined with trastuzumab in trastuzumab-sensitive and -resistant breast cancer cells (Scaltriti et al., 2011). Nonetheless, the resistance mechanism of lapatinib is considered quite different from that of trastuzumab (Wang et al., 2011), and the effects of HSP90 inhibitors on lapatinib-refractory breast cancer cells have not yet been reported.

In this study, we investigated the antitumor effects of lapatinib and ganetespib, a highly potent second-generation HSP90 inhibitor, on both lapatinib-sensitive and -resistant breast cancer cells. Initially, we established the acquired lapatinib-resistant HER2 + breast cancer cell models SKBR3-L and BT474-L, characterized by sharply increased IC_50_ values (approximately 48 and 66-fold higher than those of their parent cells) in cell proliferation and colony formation assay following treatment with lapatinib (Figures 1A,C). Ganetespib exhibited spectacular antiproliferative effects in both lapatinib-sensitive and -resistant cells, in addition to demonstrating synergistic inhibition when coupled with lapatinib (Figures 1B,C, 2A,B). Upregulation of HER3 and persistent activation of downstream PI3K/Akt and Raf/MEK/ERK have been recognized as important ways to avoid therapeutic suppression by anti-HER2 agents (Garrett et al., 2013; D'Amato et al., 2015), and acquired lapatinib-resistant cells demonstrated similar reactivation of HER signaling and downstream pathways to their parental cells (Figure 2C). However, this reactivation could be remarkably abrogated by ganetespib treatment in association with lapatinib.

Moreover, the joint administration of ganetespib and lapatinib enhanced early apoptosis and G0/G1 arrest compared to the single agents (Figure 3). Lapatinib-resistant cells escaped from early apoptosis and G0/G1 arrest at a dosage of lapatinib which could inhibit proliferation of lapatinib-sensitive cells, suggesting that dysregulation of cell cycle checkpoints and apoptosis signaling may be mechanistically involved in lapatinib resistance. The STAT3 transcription factor is considered to modulate cell growth, cell-cycle transition and apoptosis in a variety of human tumors (Wang et al., 2012). A luciferase assay used to detect STAT3 transcriptional activity demonstrated that the nuclear translocation ability of STAT3 is remarkably increased in lapatinib-resistant cells (Figure 4A). Aberrant intranuclear STAT3 has been reported to activate numerous downstream transcription factors, including Bcl-xl, Bcl-2, cyclin D1, CDK4 and c-Myc, through the inhibition of tyrosine kinase signaling (Vigneron et al., 2008). The increased nuclear transcription activity of STAT3 has also been observed in cells resistant to another TKI, erlotinib (Li et al., 2013; Lee et al., 2014). Specifically, Bcl-2/Bcl-xl is considered to be an important anti-apoptotic protein that inhibits programmed cell death (Cheng et al., 2001). Cyclin D1/CDK4 is responsible for cell progression through G1 phase, and overexpression of Cyclin D1/CDK4 can enable cells with unrepaired structural or genomic damage to traverse the G1/S checkpoint (Robles et al., 1996; Zhou and Elledge, 2000; Lee and Yang, 2003). C-Myc is also crucial in the regulation of G1/S phase proteins, and upregulation of c-Myc is commonly caused by chromosomal translocations and point mutations in cancers (Porter et al., 1994; Boxer and Dang, 2001). Decreased levels of nuclear STAT3, Bcl-2/Bcl-xl, Cyclin D1/CDK4 and c-Myc were observed in both SKBR3-L and BT474-L cells following treatment with ganetespib alone or in combination with lapatinib (Figure 4D). This disruption of the STAT3-mediated survival pathway likely reduces capacity for DNA repair and replication, in turn resulting in early apoptosis and cell cycle arrest in the G1 phase.

The therapeutic benefit of combination treatment with ganetespib and lapatinib was verified by the synergistic inhibition of tumor growth in both SKBR3 and SKBR3-L xenografts (Figure 5). Combination treatment reinforced tumor growth inhibition and enhanced suppression of HER-mediated signaling, consistent with the results obtained *in vitro*. Importantly, SKBR3-L and BT474-L cells were not entirely refractory to lapatinib treatment in cell or xenograft models (Figures 1, 5), suggesting the feasibility of lapatinib cooperating with other complementary agents to overcome lapatinib resistance.

## Conclusion

In summary, combination treatment with ganetespib and lapatinib showed synergistic inhibition of the HER and downstream PI3K/Akt and Ras/MEK/ERK pathways, in addition to enhancing induction of early apoptotic cell death and G1 arrest in both lapatinib-sensitive and -resistant breast cancer cells. Combined administration also reduced aberrant nuclear levels of the transcription factor STAT3 and components of its downstream signal pathways, likely underlying the mechanism of lapatinib resistance in HER2-positive breast cancer cells. Compared to the results of monotherapy, augmented inhibition of tumor growth was also observed in both SKBR3 and SKBR3-L xenografts following combined treatment. Our data suggest that combination treatment with ganetespib and lapatinib is a promising strategy for lapatinib-resistant HER2-positive breast cancer.

## Data Availability

The original contributions presented in the study are included in the article/Supplementary Material, further inquiries can be directed to the corresponding authors.
