# Supplementary figures and images for: Synergistic Activity of the HSP90 Inhibitor Ganetespib With Lapatinib Reverses Acquired Lapatinib Resistance in HER2-Positive Breast Cancer Cells
Source: Front Pharmacol. 2021 Jul 5;12:651516. doi: 10.3389/fphar.2021.651516 (PMC8287059; doi:10.3389/fphar.2021.651516)

## Slide 1
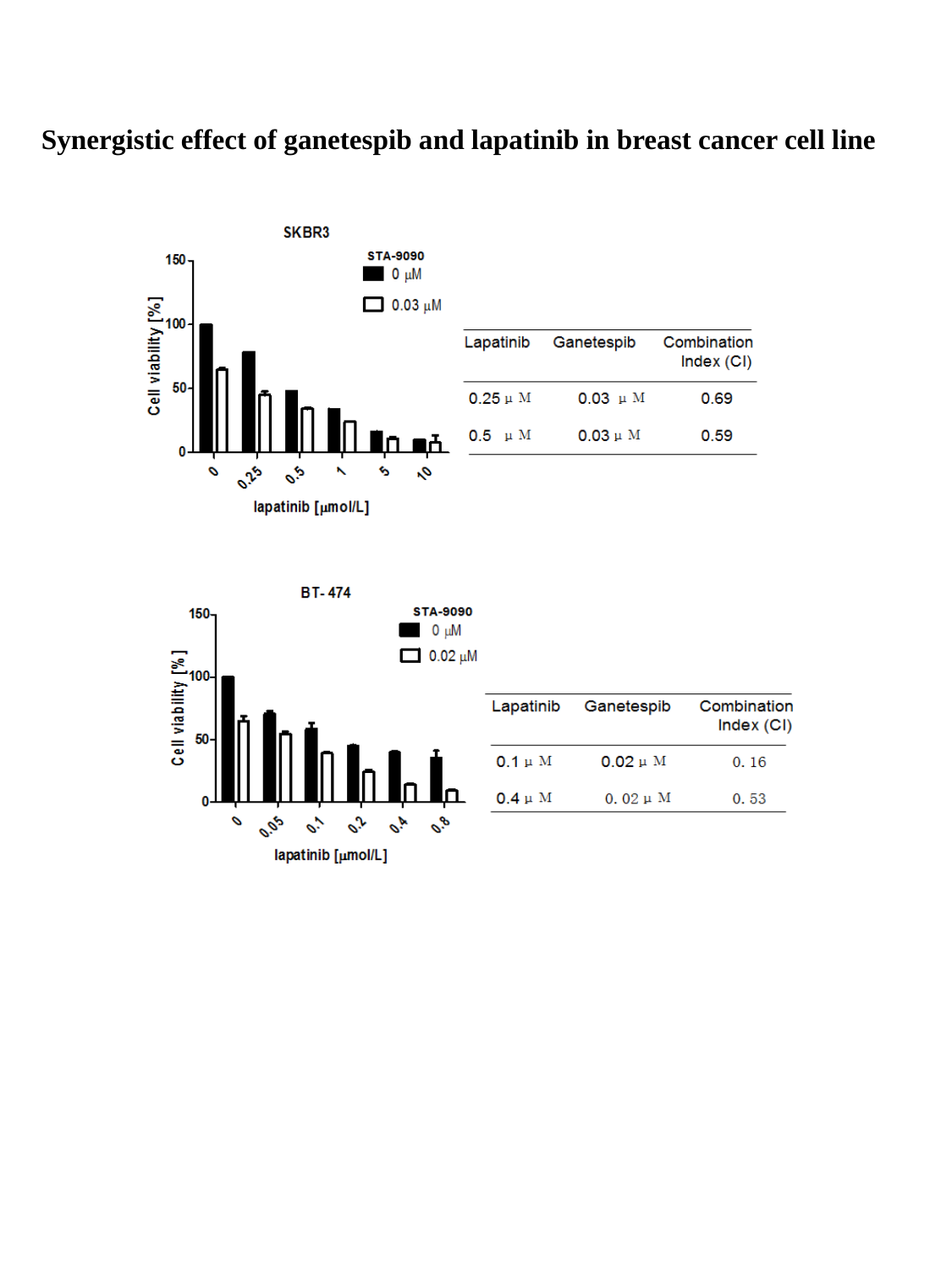

Synergistic effect of ganetespib and lapatinib in breast cancer cell line

Supplement: Supplementary file 1 [file Presentation1.PPTX]
